# Influence of Acute Inflammation on the Expression of Clock Genes in the Ovine Pars Tuberalis Under Different Photoperiodic Conditions

**DOI:** 10.3390/ijms252111471

**Published:** 2024-10-25

**Authors:** Karolina Wojtulewicz, Monika Tomczyk, Maciej Wójcik, Hanna Antushevich, Joanna Bochenek, Andrzej Przemysław Herman

**Affiliations:** Department of Genetic Engineering, The Kielanowski Institute of Animal Physiology and Nutrition, Polish Academy of Sciences, Instytucka 3, 05-110 Jabłonna, Poland; m.tomczyk@ifzz.pl (M.T.); m.wojcik@ifzz.pl (M.W.); a.antuszewicz@ifzz.pl (H.A.); j.bochenek@ifzz.pl (J.B.); a.herman@ifzz.pl (A.P.H.)

**Keywords:** clock genes, LPS, ovine, *pars tuberalis*, photoperiod

## Abstract

The *pars tuberalis* (PT) plays an important role in the photoperiodic regulation of the secretory activity of the pituitary gland. Additionally, PT secretory activity may be influenced by the animal’s immune status. The melatonin signal processing in PT cells occurs through the presence of melatonin receptors and the expression of molecular clock genes. This study aimed to define the effects of acute inflammation induced by intravenous administration of lipopolysaccharide (LPS) on the expression of clock genes in the PT of ewes under different photoperiodic conditions. Two analogous experiments were conducted in different photoperiods: short-day and long-day. Both experiments included 24 sheep divided into two groups: day (n = 12) and night (n = 12), further subdivided into a control group (n = 6) and a group treated with LPS (n = 6) at a dose of 400 ng/kg. Under short-day conditions, the expression of clock circadian regulator, basic helix-loop-helix ARNT like 1, cryptochrome circadian regulator (CRY) 1, 2, and casein kinase 1 epsilon genes was lower during inflammation. LPS injection increased expression of the period circadian regulator 1 gene during the night. Under long-day conditions, CRY1 mRNA level was lower during the night, while diurnal CRY2 mRNA expression was decreased after LPS injection. Our results showed that inflammation disturbed the expression of molecular clock genes in the PT; however, this influence was partly dependent on photoperiod conditions.

## 1. Introduction

In animals, daily and seasonal fluctuations in physiology are observed, including sleep and wakefulness rhythm, reproduction, or changes in coloration, which enable adaptation to shifting environmental conditions. The changing of night length is the primary environmental signal governing these rhythms [1]. Many animals exhibit seasonal rhythms also in their reproductive processes, where breeding activity is limited to the most optimal time of year to ensure that birth is timed to occur during optimal conditions for offspring growth and development, as well as maternal lactation support. These animals can be categorized into two groups, i.e., long-day breeders and short-day breeders [2]. The first group includes species such as ferrets, hedgehogs, hamsters, and horses, which begin the breeding season following the winter solstice. Conversely, short-day breeders, including deer, goats, and sheep, initiate their reproductive activity as daylight hours shorten, typically from late summer to early autumn [3]. At the molecular level, both seasonal and circadian rhythms are regulated by a molecular clock mechanism based on complex transcriptional and translational feedback loops (TTL) involving the so-called clock genes [4]. The activating complexes in the positive feedback loop are heterodimers formed by the circadian locomotor output cycles kaput (CLOCK) and basic helix-loop-helix ARNT-like protein 1 (BMAL) proteins. These complexes activate the expression of genes encoding period circadian regulator (PER) 1 and 2, and cryptochrome circadian regulator (CRY) 1 and 2 [5]. The resulting PER-CRY protein heterodimer is transported to the cell nucleus, where it inhibits its own gene expression by interacting with the CLOCK-BMAL complex. The degradation of the PER protein, preceded by its appropriate phosphorylation, is necessary to unblock *Per* and *Cry* gene transcription. Casein kinase 1 epsilon (CK1ε) and casein kinase 1 delta (CK1δ) are the main kinases that regulate the levels of the PER-CRY complex. They phosphorylate PER and CRY proteins, leading to their proteasomal degradation, which drives the entire cycle [4,5,6]. Light, along with the ratio of day-to-night length, serves as the primary synchronizer of the molecular clock. The light signal is transmitted to the central clock located in the suprachiasmatic nuclei (SCN) through neural pathways [7]. Melatonin in turn is the primary transmitter synchronizing clock gene expression to day/night conditions in other tissues. This indoleamine is synthesized in the dark in the pinealocytes of the pineal gland, and its quantity strictly depends on the duration of the dark phase. The necessary compound of melatonin synthesis is tryptophan, transformed by hydroxylase into 5-hydroxytryptophan, which is in turn transformed by 5-hydroxytryptophan decarboxylase into 5-hydroxytryptamine (serotonin). Further synthesis involves N-acetylation by serotonin N-acetyltransferase (its activity varies daily) to form N-acetylserotonin, which is O-methylated by 5-hydroxyindole-O-methyltransferase to form 5-methoxy-N-acetyltryptamine or melatonin [8]. It should be noted, however, that the absence of the melatonin signal does not abolish the rhythmic expression of clock genes, because the rhythm is maintained in constant conditions, i.e., without light, with a period of approximately 24 h [7,9,10,11].

Adaptation of the body to changing environmental conditions relies on the dynamic activity of numerous endocrine systems [12]. Research has shown that the production, secretion, and abundance of various endocrine factors are tightly regulated by predictable time-of-day patterns, as is the sensitivity of target organs to these signals [13]. Most mammalian species show seasonal variations in their ovulation frequency, with the activity of the hypothalamic–pituitary–gonadal (HPG) axis also exhibiting diurnal fluctuation. Reproductive processes are regulated by gonadotropin-releasing hormone (GnRH) synthesized in hypothalamic neurons and secreted into the pituitary portal circulation. Changes in the frequency and amplitude of GnRH impulses have a varied impact on the synthesis and release of luteinizing hormone (LH) and follicle-stimulating hormone (FSH) from the pituitary [14,15,16,17,18]. It has been postulated that, partially, circadian differences in gonadotropins secretion may be due to changes in GnRH release generated by the endogenous clock located in the suprachiasmatic nucleus (SCN) [19]. However, it is believed that photoperiod-dependent changes in gonadotropins secretion are influenced by melatonin action within the anterior lobe of the pituitary gland known as the *pars tuberalis* (PT). PT cells secrete hormones, including gonadotropins [20], but also express melatonin receptors (MT1), which distinguishes them from other pituitary cells [16,18]. It has been proposed that the PT regulates and supports the secretory activity of the *pars distalis* (PD) [21] and plays a crucial role as a master controller of seasonal breeding in mammals [22]. Moreover, our previous study has shown that PT cells express genes encoding several pro-inflammatory cytokines and their corresponding receptors, suggesting that this region of the pituitary gland may also be one of the areas for immune–endocrine interactions which may be dependent on light conditions [23].

It is worth mentioning that immune/inflammatory challenges activated by numerous stimuli, including bacterial endotoxin, may inhibit reproductive processes in animals, affecting the secretory activity of the HPG axis [24,25,26]. Inflammation induced by bacterial endotoxin-lipopolysaccharide (LPS) administration may decrease GnRH neuronal activity by stimulating negative inputs such as opioids and GABA. LPS administration to adult rats inhibited steroid-induced LH release and reduced Fos expression in GnRH neurons [27]. The administration of LPS has been also found to suppress the pulsatile LH release in gonadectomized rats [28,29,30]. The study on sheep also showed that endotoxin-induced inflammation could alter circulating concentrations of LH at the level of the pituitary via inhibition of LH production and release or inhibition of the LH response to GnRH stimulation [31]. Both acute and prolonged inflammation influence LH secretion in a similar manner; however, only prolonged inflammation disturbs the secretion of FSH in ewes [17,32,33]. Our previous study showed that acute inflammation also affects the melatonin action on LH secretion from the PT, which may be one of the mechanisms via which immune/inflammatory challenges disturb reproduction processes in animals [31]. This also suggests that inflammation may be an important factor influencing the functioning of PT. However, there is a lack of studies analyzing the effect of peripheral inflammation on the clock gene expression in the mammalian pituitary.

Our previous study also described diurnal and seasonal changes in basal clock gene expression in the PT of ovines. We found that both diurnal and nocturnal PT clock gene expression was higher during the short-day (SD) season compared to the long-day (LD) photoperiod. This means that protein products resulting from the expression of these genes may have a stronger effect on PT secretory activity under SD conditions [18]. The importance of the proper functioning of the biological clock in the PT for pituitary hormone secretion was underscored by the work carried out on transgenic PER1 and MT1 knockout mice, diagnosed with a reversed expression rhythm of the gene encoding the beta subunit of thyroid stimulating hormone (TSH) or a switch-off of the day–night rhythm of *TSHβ* gene expression [34]. Considering the key role of the PT in the specific integration and processing of signals from the endocrine and immune systems, as well as its involvement in the photoperiodic control of hormone secretion, it is necessary to better understand the processes occurring in this region of the pituitary gland. Therefore, this study aimed to define the influence of acute inflammation induced by intravenous administration of bacterial endotoxin on the expression of clock genes in the PT of ewes under different photoperiodic conditions.

## 2. Results

### Effect of LPS Administration on the Expression of Clock Genes in the PT

**Figure 1:**
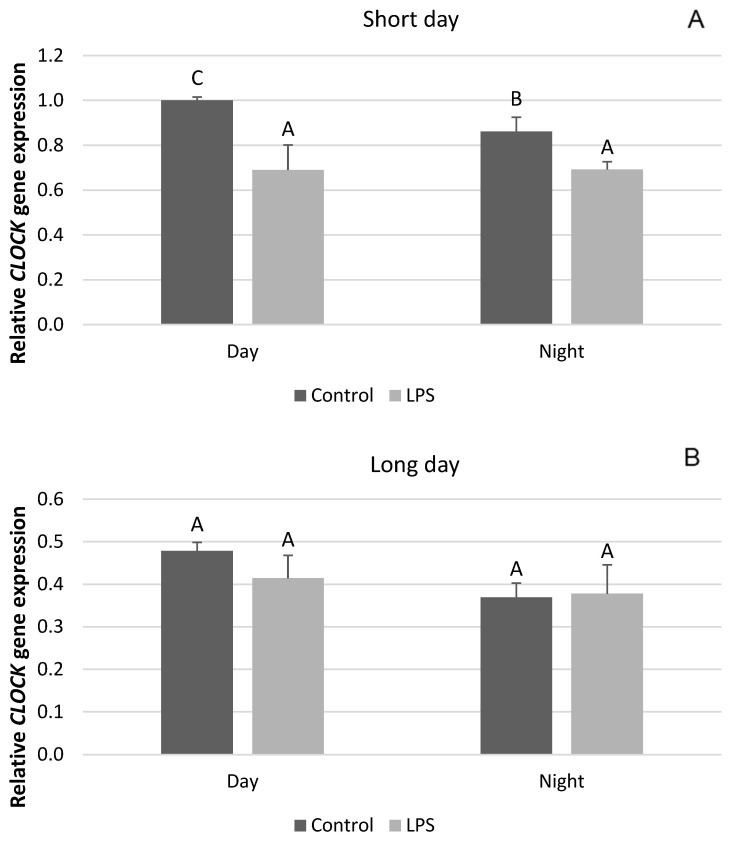
Effect of lipopolysaccharide (LPS) injection at a dose of 400 ng/kg bw on the expression of clock circadian regulator (*CLOCK*) under short-day (SD) (**A**) and long-day (LD) (**B**) conditions during the day and night. Data are presented as means ± SEM; significant differences marked with different capital letters above the bars were analyzed by a two-way ANOVA followed by a Fisher’s post hoc test. Statistical significance was stated when (*p* < 0.05). Values marked with the same capital letters do not differ significantly.

**Figure 2:**
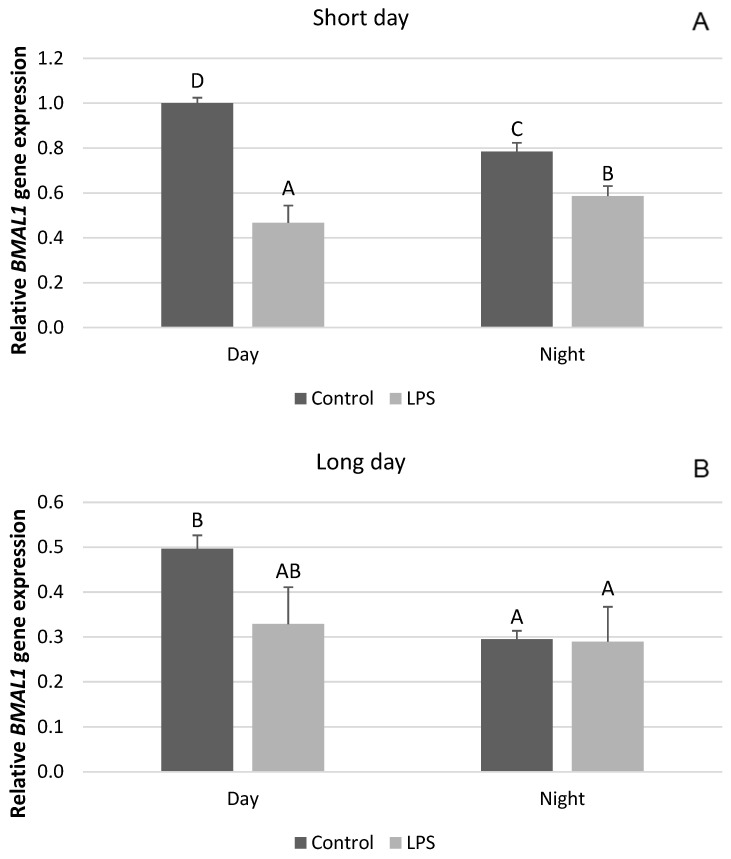
Effect of lipopolysaccharide (LPS) injection at a dose of 400 ng/kg bw on the expression of clock circadian regulator (*BMAL1*) under short-day (SD) (**A**) and long-day (LD) (**B**) conditions during the day and night. Data are presented as means ± SEM; significant differences marked with different capital letters above the bars were analyzed by a two-way ANOVA followed by a Fisher’s post hoc test. Statistical significance was stated when (*p* < 0.05). Values marked with the same capital letters do not differ significantly.

**Figure 3:**
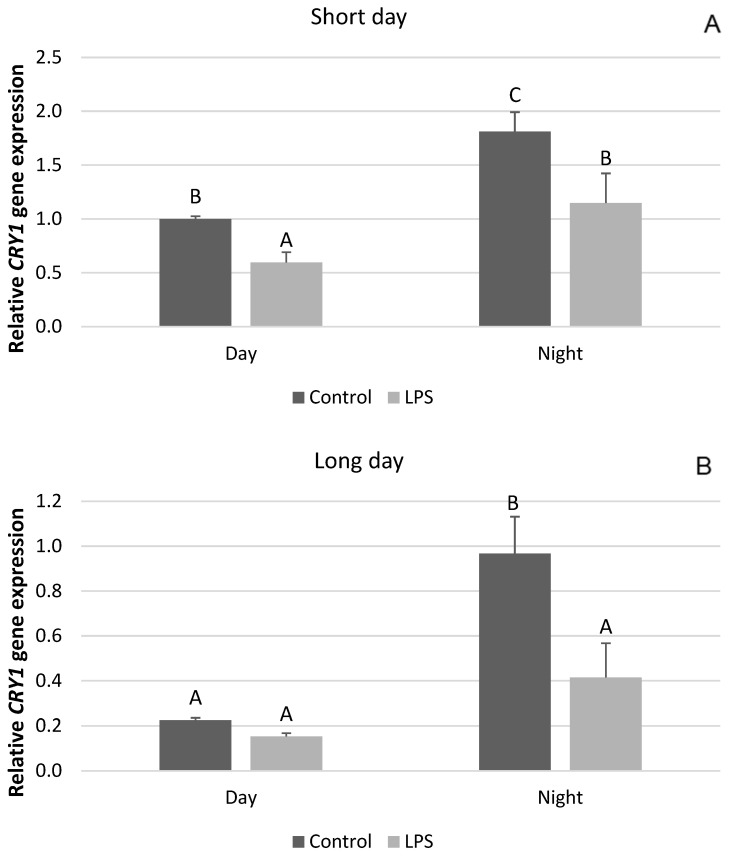
Effect of lipopolysaccharide (LPS) injection at a dose of 400 ng/kg bw on the expression of clock circadian regulator (*CRY1*) under short-day (SD) (**A**) and long-day (LD) (**B**) conditions during the day and night. Data are presented as means ± SEM; significant differences marked with different capital letters above the bars were analyzed by a two-way ANOVA followed by a Fisher’s post hoc test. Statistical significance was stated when (*p* < 0.05). Values marked with the same capital letters do not differ significantly.

The influence of inflammation on the expression of clock genes in the PT depended upon the photoperiodic conditions. Endotoxin treatment reduced (*p* < 0.05) the level of mRNA for *CLOCK* both during the day and at night in the SD season (Figure 1A). On the other hand, the administration of LPS did not influence the gene expression of *CLOCK* during LD (Figure 1B). The expression of the *BMAL* gene was also reduced (*p* < 0.05) by LPS administered in SD conditions (Figure 2A), while in the LD photoperiod, the level of this gene expression did not change (Figure 2B). Inflammation lowered (*p* < 0.05) the level of *CRY1* gene expression under SD conditions both during the day and at night (Figure 3A), whereas during LD, endotoxin injection inhibited (*p* < 0.05) only the nocturnal expression of this gene (Figure 3B).

**Figure 4:**
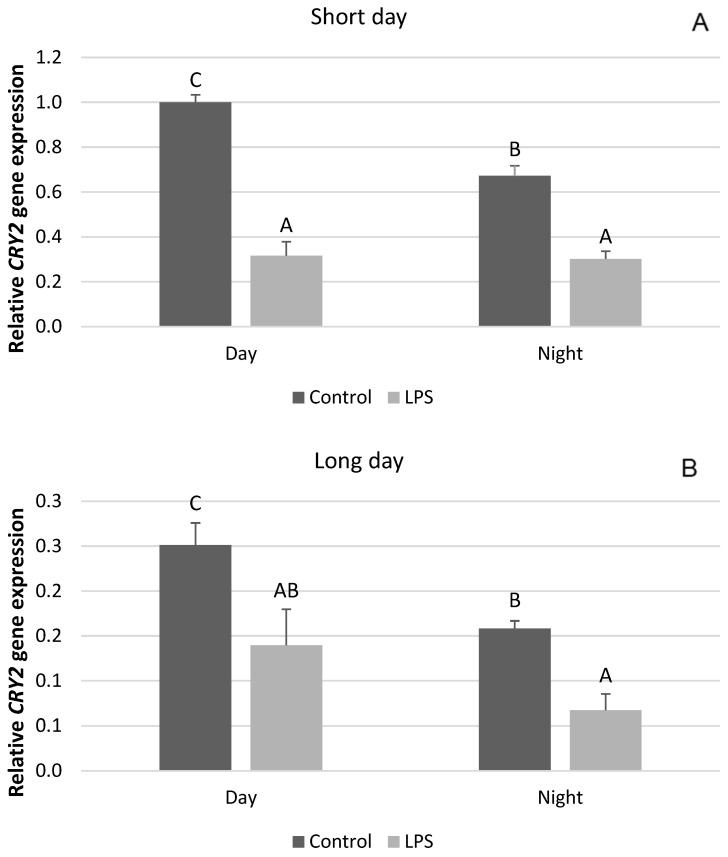
Effect of lipopolysaccharide (LPS) injection at a dose of 400 ng/kg bw on the expression of clock circadian regulator (*CRY2*) under short-day (SD) (**A**) and long-day (LD) (**B**) conditions during the day and night. Data are presented as means ± SEM; significant differences marked with different capital letters above the bars were analyzed by a two-way ANOVA followed by a Fisher’s post hoc test. Statistical significance was stated when (*p* < 0.05). Values marked with the same capital letters do not differ significantly.

**Figure 5:**
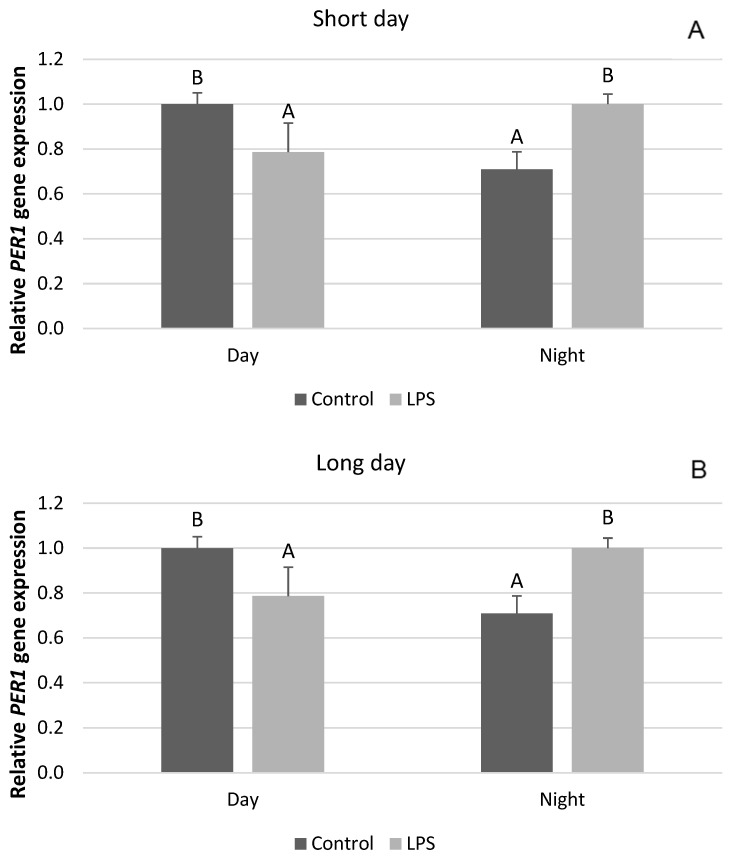
Effect of lipopolysaccharide (LPS) injection at a dose of 400 ng/kg bw on the expression of clock circadian regulator (*PER1*) under short-day (SD) (**A**) and long-day (LD) (**B**) conditions during the day and night. Data are presented as means ± SEM; significant differences marked with different capital letters above the bars were analyzed by a two-way ANOVA followed by a Fisher’s post hoc test. Statistical significance was stated when (*p* < 0.05). Values marked with the same capital letters do not differ significantly.

**Figure 6:**
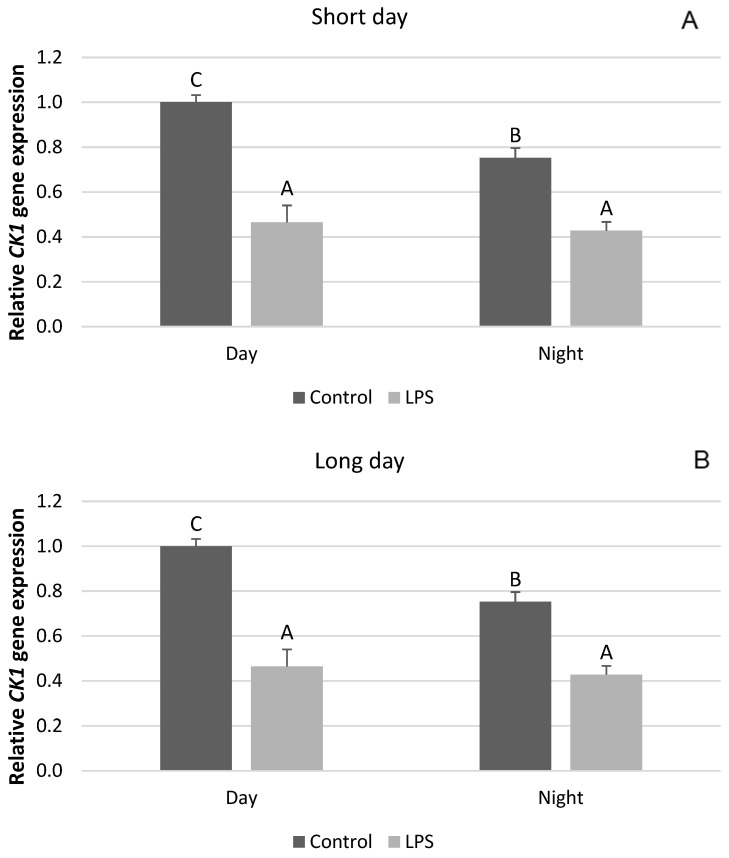
Effect of lipopolysaccharide (LPS) injection at a dose of 400 ng/kg bw on the expression of clock circadian regulator (*CK1*) under short-day (SD) (**A**) and long-day (LD) (**B**) conditions during the day and night. Data are presented as means ± SEM; significant differences marked with different capital letters above the bars were analyzed by a two-way ANOVA followed by a Fisher’s post hoc test. Statistical significance was stated when (*p* < 0.05). Values marked with the same capital letters do not differ significantly.

Interestingly, our research showed that, unlike the clock genes mentioned, there are no seasonal differences in the effect of acute inflammation of the gene expression of *CRY2*, *PER2*, and *CK1*. It was found that the administration of LPS decreased (*p* < 0.05) the diurnal and nocturnal expression of the *CRY2* gene. This effect was observed both in SD (Figure 4A) and LD conditions (Figure 4B). Endotoxin administered under SD conditions decreased (*p* < 0.05) the diurnal expression of the *PER1* mRNA, while it increased (*p* < 0.05) the nocturnal expression of this gene (Figure 5A). The same effect of LPS-induced inflammation on the level of *PER2* mRNA was demonstrated under the LD photoperiod (Figure 5B). The administration of endotoxin in SD (Figure 6A) and LD (Figure 6B) conditions lowered (*p* < 0.05) the level of mRNA for CK1 both during the day and at night.

## 3. Discussion

The results support previous findings indicating circadian fluctuation in the clock gene mRNA level in the PT [18,33]. On the other hand, it should be noted that in previous works, the trend of circadian changes in the expression of clock genes remained relatively constant, irrespective of the season analyzed [18]. The present study demonstrated a predominantly inhibitory effect of acute inflammation induced by bacterial endotoxin injection on the expression of molecular clock genes in the PT, but only under SD conditions. The only exception was the *PER1* gene, whose expression was reduced by LPS administration during the day, while at night, endotoxin-treated animals showed increased *PER1* mRNA expression in the PT. Meanwhile, the impact of LPS administration on clock gene expression under LD conditions was more variable, with reductions observed in daytime *BMAL* and *CRY2* gene expression, and in night-time *CRY2* gene expression.

Our findings in the sheep model demonstrated that acute immune stress induced by endotoxin injection affected the expression of certain clock genes in the PT. These results are consistent with previous studies on rodents showing that intraperitoneal administration of LPS-induced photic-like phase delays in mice [35], or suppression of biological clock genes in male Wistar rats caused by intravenous LPS [36]. Moreover, another mouse study revealed that LPS injection suppressed the amplitude of *Per2* expression in the ovary, while also inhibiting the expression of cytochrome P450 aromatase (CYP19) and luteinizing hormone receptor (LHr) genes on the ovary of mice treated with equine chorionic gonadotropin (eCG). This suggests that the inflammation-dependent inhibition of *Per2* expression may be associated with the inhibition of CYP19 and LHr expression by LPS in the ovaries of immature mice [37]. These findings collectively imply that immune system activation should be regarded as a stimulating signal for circadian clock gene expression both in nocturnal and diurnal animals. Although the expression patterns of most clock genes are similar in both nocturnal and diurnal species [38], our study, demonstrating that inflammation may disturb the expression of clock genes in the PT, may be more representative of diurnal animals. It should be noted that dysregulation of clock gene expression could have a significant impact on animal health status, including immune system function. Among these genes, *BMAL1* seems to play the most important role in the immune response. Previous research demonstrated that mice with *Bmal1* deletion lacked a circadian rhythm in the levels of pro-inflammatory cytokines (especially IL-6) during endotoxin-induced inflammation [39,40]. *BMAL1* was also found to play a significant role in the development of B cells. In mice with the *Bmal1*^−/−^ genotype, a reduced number of mature B cells was present in the peripheral blood, bone marrow, and spleen [41]. Moreover, in a model of *Listeria monocytogenes* infection, *Bmal1* was shown to regulate the circadian rhythm of monocyte recruitment to tissues [41,42]. *CLOCK* is another gene whose activity is important for the functioning of the inflammatory response. A reduced inflammatory response to endotoxin was found to occur in *Clock* knockout mice [43]. Decreased activity of nuclear factor kappa-light-chain-enhancer of activated B cells (NF-κB) was demonstrated in Clock-deficient animals, whereas NF-κB activation was directly proportional to *Clock* overexpression. Importantly, the same study showed an inverse relationship between NF-κB activation and *Bmal1* gene expression [44]. Studies have reported that PER and CRY proteins play crucial roles in the control of cancer cell development. In mice, *Per1* and *Per2* were identified as tumor suppressors, while PER2-deficient animals had an increased risk of genetic and UV-induced cancer [45]. Conversely, overexpression of both *Per* genes was shown to inhibit cancer cell growth in vivo and promote apoptosis in vitro [45]. Interestingly, mice lacking both p53 and CRY protein expression did not develop cancers and had a longer lifespan compared to those deficient in p53 alone [46]. Reduced levels of CRY1/CRY2 proteins were also found to promote cellular apoptosis in animals exposed to UV radiation [47,48,49]. Thus, CRY deficiency and PER overexpression appear to act similarly in inhibiting tumor growth, suggesting that disruption of circadian genes during aging involves a complex interplay between their roles in the circadian clock and other cellular processes and pathways [50]. Importantly, accumulating evidence from human studies suggests that modern lifestyles, which significantly disturb the body’s natural circadian rhythms, may consequently lead to disruptions in reproductive processes [51], increased cancer risk [52,53], cardiovascular diseases [54,55], and many other health problems.

The proper rhythmic changes in the expression of biological clock genes may regulate the secretory activity of numerous glands. A recent study on rats aimed to determine the role of clock genes in the endocrine function of the pineal gland, particularly focusing on the transcription of the alkylamine N-acetyltransferase (Aanat) gene encoding the enzyme responsible for generating the rhythm of melatonin synthesis. The study showed that knocking down the *Clock* gene resulted in a marked overexpression of *Aanat* in pinealocytes, suggesting that the expression of this gene in these cells regulates the diurnal profile of *Aanat* expression [56]. Another study on mice provided evidence supporting the critical role of clock genes in the control of tear secretion in the lacrimal gland. Diurnal and circadian rhythms were demonstrated to occur during tear secretion in wild-type mice, with tear volume increased in the objective and subjective night. Conversely, disruption in diurnal and circadian rhythms of tear secretion was observed in mice deficient in core clock genes (*Cry1*^−/−^ *Cry2*^−/−^) [57].

The proper functioning of the biological clock also appears to be important for the function of the pituitary gland. An in vitro study revealed a correlation of the expression of *Per2* and *Clock* with proopiomelanocortin (POMC) and prolactin in pituitary cells. Knockdown experiments targeting *Per2* and *Clock* resulted in the suppression of POMC and PRL expression, respectively [58]. Moreover, the latter study suggested that this effect could be partially induced by melatonin which inhibited *Per2* expression in corticotroph cells, but did not affect *Clock* in lactotroph cells [58]. This implied that the clock gene could be involved in the melatonin-dependent modulation of certain pituitary hormone synthesis pathways. The PT is a part of the pituitary gland involved in the photoperiodic regulation of hormone secretion because cells located in this area express melatonin receptors [21,59]. This in turn enables melatonin to influence the activity of PT cells and may explain day–night differences in clock gene expression found in our study. It should be noted that our previous ex vivo studies suggested an important role of the PT in the secretion of LH, as they demonstrated that the basal and GnRH-stimulated secretion of LH in the PT explants was even higher than that determined in anterior pituitary explants [60,61]. Additionally, it was found that the stimulatory effect of GnRH on LH release could be abolished by melatonin administration [60]. Daily changes in the circulating concentration of melatonin play an important role in the photoperiodic regulation of gonadotropins, particularly LH levels. Studies confirm that in sheep, both the level of LH mRNA and the concentration of this hormone in the blood are higher during the day. Moreover, these values are higher in animals in the follicular phase compared to animals in the anestrus phase [16]. It is noteworthy that studies on mice gonadotrophs showed the presence of a functional molecular clock in these cells. A conditional deletion of Bmal1 in this cell population was found to cause only a modest increase in LH levels in proestrus, and increased FSH levels during estrus, resulting in increased variability in the estrus cycle, but no impact on fertility. This data suggests that the intrinsic clock in gonadotrophs is dispensable for LH surge regulation, but contributes to the stability of the estrus cycle [62]. An in vitro study on mouse gonadotropin LβT2 cells revealed that clock genes and the bone morphogenetic protein (BMP) system could be involved in the regulation of gonadotropin secretion by GnRH. The latter authors demonstrated that LH expression in gonadotropin LβT2 cells exhibited Bmal1/Clock-dependent fluctuations under the influence of GnRH. After GnRH stimulation, these fluctuations were modulated in a phase-dependent manner by extracellular signal-regulated kinases (ERKs) and bone morphogenetic proteins (BMPs) in the early and late stages, respectively [63].

## 4. Materials and Methods

### 4.1. Experimental Design

**Figure 7:**
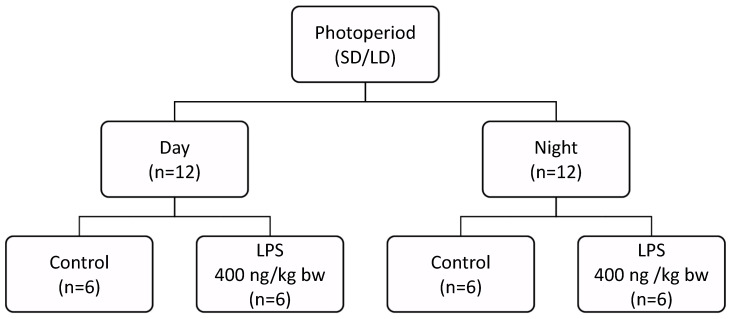
Schematic of the experimental set-up carried out analogously under short-day (SD, 8:16) and long-day (LD, 16:8) conditions. In each experiment (n = 24), animals were divided into day (n = 12) and night groups (n = 12), with subgroups formed within each group–control (n = 6) and lipopolysaccharide-treated (LPS; 400 ng/kg bw; iv.; n = 6) animals.

Two analogous experiments were conducted at two different photoperiods that involved 2–3-year-old ewes. The experiments were carried out one month before the winter solstice (short-day; SD; 8:16) and the summer solstice (long-day; LD; 16:8), respectively. In the SD photoperiod, the estrus cycle was synchronized using a Chronogest^®^ CR (Merck & Co., Inc., Rahway, NJ, USA) following the methodology described in [64,65], to minimize the impact of different levels of gonadal steroids on the inflammatory-dependent response of the expression of clock gene. The experiments were performed during the 10 days of the luteal phase, coinciding with estradiol and progesterone plateau. Each experiment included 24 sheep allocated to two groups: day (n = 12) and night (n = 12), with a control subgroup (n = 6) and an experimental group treated with lipopolysaccharide (LPS) (n = 6) (Figure 7). The animals were housed indoors under natural lighting conditions (52° N, 21° E) and had constant access to food according to the recommendation of the National Research Institute of Animal Production for adult ewes [66]. The ewes had visual contact with each other to avoid isolation stress. Three hours before midday or midnight, an appropriate volume of LPS isolated from *E. coli* 055:B5 (400 ng/kg BW) (Merck KGaA, Darmstadt, Germany), dissolved in saline (0.9% *w*/*v* NaCl; Baxter, Deerfield, IL, USA), was injected intravenously (iv.) into the jugular vein using an intrajugular catheter (implanted the day before the experiment). The maximum volume of the injected LPS solution never exceeded 2.5 mL. The control group was administered the same volume of NaCl based on their body weight. The efficiency of the LPS treatment in inducing an inflammatory response was assessed by measuring body temperature (from 39.1 °C ± 0.3 to ±41.0 °C ± 0.2). All procedures in the night experiment were performed under red light. Animal euthanasia was performed at midday or midnight. Sheep were slaughtered after prior pharmacological stunning (xylazine 0.2 mg/kg of body mass and ketamine: 3 mg/kg of body mass, iv.) according to the method described elsewhere [67], and the brains were promptly removed from the skulls; the *PT* was dissected, immediately frozen in liquid nitrogen, and stored at −80 °C until further analysis.

All procedures on animals were carried out with the approval of the Local Ethics Committee of Warsaw University of Life Sciences at SGGW (Warsaw, Poland), authorized under No. 23/2015 (approved on 21 May 2015) and No. WAW2/62/2017 (approved on 21 June 2017).

### 4.2. Analysis of Relative Gene Expression by RT-qPCR

Total RNA isolation from PT was performed by the NucleoSpin^®^ RNA kit (MACHEREY-NAGEL GmbH and Co. KG, Düren, Germany). All isolation steps were conducted following the manufacturer’s instructions. The purity and integrity of the obtained RNA were confirmed according to the method described in the publication [60]. For cDNA synthesis, the Maxima™ First Strand cDNA Synthesis Kit for RT-qPCR (Thermo Fisher Scientific, Waltham, MA, USA) was used. The real-time PCR reactions were conducted on a Rotor-Gene Q machine (Qiagen, Dusseldorf, Germany) using HOT FIREPol EvaGreen^®^ qPCR Mix Plus (Solis BioDyne, Tartu, Estonia) and specific oligonucleotide primers (Genomed, Warsaw, Poland). The primers used for analysis were selected based on our previous study or originally designed using the Primer3 version 4.1.00 (The Whitehead Institute, Boston, MA, USA) bioinformatic tool (Table 1). Amplification specificity was confirmed by the final melting curve analysis.

Relative gene expression analysis was performed using Rotor-Gene Q Series Software 1.7 (Qiagen GmbH, Hilden, Germany) according to a previous study [18]. Gene expression values were normalized to the average relative level of determined mRNA in the control group from the SD photoperiod, which was set to 1.0.

### 4.3. Statistical Analysis

The obtained results were analyzed with a two-way (two parameters: day/night and LPS) analysis of variance (ANOVA) and then with Fisher’s post hoc test in the STATISTICA 12 software (Stat Soft. Inc., Tulsa, OK, USA). Data are presented as means ± SEM, with statistical significance set at *p* < 0.05.

## 5. Conclusions

Our research demonstrated that endotoxin-induced inflammation disrupted the expression of molecular clock genes in the ovine PT, but this effect was partly dependent on photoperiodic conditions. We found that there are important seasonal differences in the effect of inflammation on the expression of CLOCK, BMAL, and CRY2 in the ovine PT. On the other hand, acute inflammation influences the expression of CRY2, PER2, and CK1 genes in the PT in a season-independent manner. Given the importance of clock genes for PT secretory activity, such disturbances may have a significant impact on the function of the anterior pituitary gland, subsequently affecting the endocrine system. A better understanding of the mechanisms through which inflammation interferes with endocrine activity may be valuable for both human and veterinary medicine. However, a more thorough elucidation of the PT’s role in the processing of photoperiodic and immunological signals requires further in-depth research.

## Figures and Tables

**Table 1:** Genes analyzed by real-time PCR: full names and abbreviations.

GenBank Acc. No.	Gene	Amplicon Size [bp]	Forward/Reverse	Sequence 5′→3′	Reference
NM_001009284.2	*B2M* beta-2 microglobulin	119	Forward	CTTCTGTCCCACGCTGAGTT	Originally designed
			Reverse	GGTGCTGCTTAGAGGTCTCG	
U39357	*ACTB* actin beta	168	Forward	CTTCCTTCCTGGGCATGG	[68]
			Reverse	GGGCAGTGATCTCTTTCTGC	
NM_001034034	*GAPDH* glyceraldehyde-3-phosphate dehydrogenase	134	Forward	AGAAGGCTGGGGCTCACT	[68]
			Reverse	GGCATTGCTGACAATCTTGA	
NM_001130932.1	*CLOCK* clock circadian regulator	115	Forward	CAGTCAGTCTCAAGGAAGCGT	Originally designed
			Reverse	GGTGTAGAGGAAGGGTCCGA	
NM_001129734.1	*BMAL* Basic helix-loop-helix-ARNT like 1	92	Forward	CGGAGTCGGTGGTTCAGTTT	Originally designed
			Reverse	TCCAGGACGTTGGCTAAAACA	
NM_001129735.1	*CRY1* cryptochrome circadian regulator 1	150	Forward	TAGCAGCAGTGCAAGTTGT	Originally designed
			Reverse	TGCGTGTCCTCTTCCTGACTA	
NM_001129736.1	*CRY2* cryptochrome circadian regulator 2	139	Forward	GATTGCGGCTCCATGACAAC	Originally designed
			Reverse	GACTGAAGCAGGAACCTCCA	
XM_027974927.1	*PER1* period circadian regulator 1	138	Forward	GTCCCTGCTACTGGCACATT	Originally designed
			Reverse	GGAGCAACTGGAGGCTTCTT	
NM_001078109.1	*CK1ε* casein kinase 1 epsilon	143	Forward	CACCTGGGCATCGAGCAAA	Originally designed
			Reverse	TTCTTCTCGCTGATCCGCTC	

## Data Availability

The raw data supporting the conclusions of this article will be made available by the authors on request.
